# Screening for Rheumatic Heart Disease among Peruvian Children: A Two-Stage Sampling Observational Study

**DOI:** 10.1371/journal.pone.0133004

**Published:** 2015-07-24

**Authors:** Ernest Spitzer, Jorge Mercado, Fabian Islas, Martina Rothenbühler, Reto Kurmann, Fabian Zürcher, Peter Krähenmann, Nassip Llerena, Peter Jüni, Pedro Torres, Thomas Pilgrim

**Affiliations:** 1 Department of Cardiology, Bern University Hospital, Bern, Switzerland; 2 Institute of Cardiology CardioSalud, Arequipa, Peru; 3 University Hospital San Carlos, Madrid, Spain; 4 Institute of Social and Preventive Medicine and Clinical Trials Unit, University of Bern, Bern, Switzerland; 5 National Hospital Carlos Alberto Seguín Escobedo, Arequipa, Peru; 6 Institute of Primary Health Care (BIHAM), University of Bern, Bern, Switzerland; University of Bologna, ITALY

## Abstract

**Background:**

The objective of the study was to evaluate the implications of different classifications of rheumatic heart disease on estimated prevalence, and to systematically assess the importance of incidental findings from echocardiographic screening among schoolchildren in Peru.

**Methods:**

We performed a cluster randomized observational survey using portable echocardiography among schoolchildren aged 5 to 16 years from randomly selected public and private schools in Arequipa, Peru. Rheumatic heart disease was defined according to the modified World Health Organization (WHO) criteria and the World Heart Federation (WHF) criteria.

**Findings:**

Among 1395 eligible students from 40 classes and 20 schools, 1023 (73%) participated in the present survey. The median age of the children was 11 years (interquartile range [IQR] 8–13 years) and 50% were girls. Prevalence of possible, probable and definite rheumatic heart disease according to the modified WHO criteria amounted to 19.7/1000 children and ranged from 10.2/1000 among children 5 to 8 years of age to 39.8/1000 among children 13 to 16 years of age; the prevalence of borderline/definite rheumatic heart disease according to the WHF criteria was 3.9/1000 children. 21 children (2.1%) were found to have congenital heart disease, 8 of which were referred for percutaneous or surgical intervention.

**Conclusions:**

Prevalence of RHD in Peru was considerably lower compared to endemic regions in sub-Saharan Africa, southeast Asia, and Oceania; and paralleled by a comparable number of undetected congenital heart disease. Strategies to address collateral findings from echocardiographic screening are necessary in the setup of active surveillance programs for RHD.

**Trial Registration:**

ClinicalTrials.gov identifier: NCT02353663

## Introduction

Rheumatic fever results from an abnormal autoimmune response to group A streptococcal pharyngitis and may progress to rheumatic heart disease (RHD) with cumulative exposure. Largely eliminated in high-income countries, RHD continues to be endemic in less privileged regions of the world where it accounts for up to a quarter million premature deaths every year [1][2].

Screening echocardiography for early detection of clinically silent rheumatic heart disease (RHD) has been recommended in endemic regions of the world, where RHD accounts for up to a quarter of a million deaths every year.[3] [1] Timely installation of secondary antibiotic prophylaxis may prevent progression of subclinical lesions to severe valvular damage and heart failure mediated by cumulative exposure to streptococcal antigens.

Differences in the criteria for the diagnosis of RHD contribute to the large heterogeneity in reported prevalence across different geographical regions, and may translate into a differential in timing of initiation of secondary antibiotic prophylaxis. Valvular lesions consistent with RHD progress along a spectrum of disease and involve isolated morphological changes of the valvular apparatus, functional lesions, or a combination of both. The prognostic significance of the individual stages needs to be determined in prospective studies of the natural history of subclinical valvular disease.

Moreover, the implications of population-based echocardiographic screening reach beyond the detection of RHD. Several studies using echocardiographic screening reported incidental findings of congenital heart disease. The latter may have repercussions on medical management and prognosis that have not been systematically assessed to date. In resource-limited settings high prevalence rates of RHD are paralleled by constrained access to healthcare, and regular medical examinations of children and adolescents are few. The importance of collateral findings from active surveillance for RHD on prognosis and healthcare expenditures in emerging countries may be underestimated.

The objective of the present analysis was to (1) evaluate the implications of different classifications of RHD on estimated prevalence, and (2) to systematically assess the importance of incidental findings from echocardiographic screening among schoolchildren in Peru.

## Methods

### Study design and patient population

We performed a cross-sectional survey of RHD among school-going children in Arequipa. Peru has a Human Development Index (HDI) of 0.737 and ranks at 82 among all 187 listed countries in the Human Development Report issued by the United Nations Development Programme[4]. Arequipa is situated at 2328 meters altitude above sea level and links the coastal and highland regions of southern Peru. We identified 457 primary and secondary schools. Using a multistage sample strategy we selected 40 classes from 20 schools for the present survey taking into account administration of the schools (governmental versus private) and the location in an urban or rural setting, respectively. Two classes per school were selected. All children from a selected class between 5 and 16 of years of age were eligible for potential inclusion. No exclusion criteria applied.

### Ethics statement

Ethical approval was obtained from the Human Research Ethics Committee of the University San Martín de Porres, Lima, Peru (Officio No. 48-2014-CIEI-USMP-CCM). Local authorizations were granted by the Regional Administrations of the Health and Education Ministries, as well as from each school’s principal. Written consent was obtained from parents/guardians, and written assent from all children. The study was registered with ClinicalTrials.gov (Identifier: NCT02353663).

### Data acquisition and echocardiographic evaluation

All selected schools were contacted and visited to inform the major stakeholders, outline the objective of the project, and gather informed consent. Subsequently, all selected classes were visited at least twice by a team of two physicians and one study nurse in order to reduce the number of absentees to a minimum. Demographic and socio-economic characteristics were acquired in a standardized interview by means of a questionnaire customized to the age of the children. A focused medical history was followed by a brief physical examination. Cardiac auscultation and echocardiographic screening were performed by two independent physicians blinded to the findings of each other. Echocardiography was performed by a specifically trained cardiologist following a structured acquisition protocol according to the WHF recommendations for acquisition[5]. A complete two-dimensional echocardiography with parasternal, apical, subcostal and suprasternal views, and M-mode and Doppler imaging was used for diagnosis (S1 Fig). A MyLabAlpha (Esaote, Italy) portable echocardiographic device and MyLabDesk^3^ (Esaote, Italy) software were utilized. Subjects with pathologic findings were re-scheduled for a detailed clinical and echocardiographic evaluation by a local cardiologist. Children with echocardiographic findings compatible with borderline or definite RHD according to the WHF criteria, or probable or definite RHD according to the WHO criteria, were invited to initiate secondary prophylaxis and regular follow-up. Patients with other diagnoses (e.g. congenital heart disease) were given professional advice according to the need of follow-up, medical or invasive treatment, and referred to the local health providers. Stored clips of all subjects were further evaluated in the echocardiographic corelab at Bern University Hospital, and measurements required for the WHF and WHO criteria were re-assessed by 5 cardiologists. Disagreements in the assessment of criteria used for diagnosis and categorization of RHD were resolved by discussion; a final diagnosis was reached after mutual agreement between reviewers or a final decision by a third party. All clips and still frames were captured and analysed using harmonics. Data was entered into a dedicated database held at the Institute of Social and Preventive Medicine at the University of Bern, Switzerland. Central data monitoring was performed. We assessed the inter-rater reliability of the echocardiographic measurements in a subset of 120 screening echocardiograms using 6 raters.

### Definitions

Acute rheumatic fever (ARF) was defined according to the Jones criteria.[6] RHD was defined according to the modified criteria of the WHO and the criteria of the WHF for individuals <20 years of age (S1 Table). In brief, the modified WHO criteria take into account both cardiac murmurs consistent with any combination of mitral regurgitation or aortic regurgitation and morphological or functional findings on echocardiography. In contrast, the criteria suggested by the World Heart Federation (WHF) rely on echocardiographic findings only and require a combination of morphological and functional criteria[5]. The presence of a pathologic heart murmur on cardiac auscultation differentiates between clinically manifest and silent disease.

### Statistical analysis

Sample size calculations were based on reported prevalence rates of RHD among school children in endemic countries using echocardiographic screening. We calculated a sample size of 1000 school children aged 5 to 16, accounting for a type I error of 0.05 and a statistical power of 80%.

The sampling was performed on the basis of the strategy applied in the international educational surveys TIMSS and PIRLS, which suggests to use a two-stage stratified sampling procedure.[7] Using this sampling procedure, we selected 40 classes. In the first sampling stage, 20 schools were sampled with probabilities proportional to their size from a list of all schools in the region of interest. The schools were stratified according to the type of school (public/private and primary/secondary) as well as their degree of urbanity (rural/urban). With the aim of ensuring adequate representation of these stratification variables, we opted for explicit stratification leading to a split of the sampling frame and thus resulting in eight smaller sampling frames. The sampling was performed within each of these sampling frames in order to ensure the desired level of precision in each stratum. The second sampling stage consisted of a random selection of two classes from each selected schools. The sampling weights account for the described sampling strategy and include adjustment to non-response at individual level.

Data is presented as counts and percentages for binary and categorical variables and as means and standard deviations (SD) or medians and interquartile ranges (IQR) for continuous variables. Height, weight and body mass index (BMI) are reported as corresponding z-scores for age and were calculated using the application tools made available by the WHO.[8] We estimated the overall prevalence rate of RHD and also calculated age-specific prevalence rates. The prevalence rates are reported by indicating the 95% confidence interval. The inter-rater reliability was assessed individually between the on-site rater and five off-site raters, using the prevalence adjusted and bias adjusted Kappa statistic to account for the poor reliability of the Kappa statistic in case of very low or high prevalence.[9] We assessed possible predictors of RHD using univariate logistic regression. We performed both an unweighted and weighted analysis to account for the effect of the sampling strategy on the estimation of the overall prevalence of RHD. The association between age groups and disease was further assessed using the χ^2^ test and the association between disease progression and age using ordered logistic regression. All analyses were performed using Stata 13 (StataCorp, College Station, TX, USA).

### Role of funding source

The study was supported by research grants of the UBS Optimus Foundation and the Gottfried and Julia Bangerter-Rhyner Foundation. The funder of the study had no role in study design, data collection, data analysis, data interpretation, or writing of the manuscript. ES and TP had access to all data in the study and had final responsibility for the decision to submit for publication.

## Results

**Fig 1:**
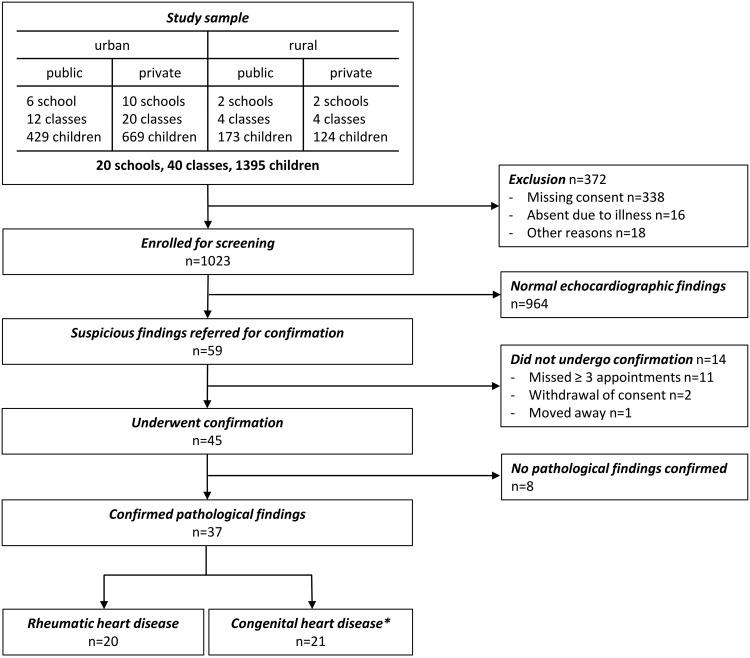
Patient flow according to STROBE statement. *4 children had both rheumatic heart disease and congenital heart disease.

Between April and May 2014 a total of 1395 children from 40 classes in 20 randomly selected schools were invited to undergo active surveillance for rheumatic heart disease. After orientation of the children and their primary caregivers, 1023 children (73%) between the ages of 5 to 16 years underwent evaluation according to a multistage screening protocol (Fig 1). Detailed information of the sampling frame and the study sample are provided in the S2 Table.

Socio-demographic characteristics are summarized in Table 1. The median age of the children was 11 years (IQR 8–13 years) and 50% were girls. More than four in five children (82%) were raised in an urban environment, and more than half of the children went to private schools (57%). The majority of the children (99%) had relatively close access to healthcare facilities (≤10 km) and reported a visit to the doctor within the last six months in 59% of the cases. 394 children (39%) reported a past medical history of a sore throat (Table 2). Two children reported migrating arthritis and one child had chorea; however, none of the children qualified for a diagnosis of ARF according to the Jones criteria. On cardiac auscultation, 44 children (4.3%) were found to have a heart murmur.

**Table 1:** Socio-demographic characteristics.

***Age*, *years (median [IQR])***	11 (8–13)
***Males (%)***	510 (49.9)
***Socio-economic criteria***	
*Number of rooms*	
≤3 rooms	137 (13.4)
4–6 rooms	427 (41.7)
≥7 rooms	459 (44.9)
*Number of family members*	
Adults per household	3 (2–4)
Children per household	2 (2–3)
Overcrowding (≥4 persons per room)	6 (0.6)
*Possessions*	
≥1 car	471 (46.0)
≥1 motorbike	113 (11.0)
≥1 television	1019 (99.6)
≥1 cellular phone	1005 (98.2)
Internet connection	598 (58.5)
≥10 books	757 (74.0)
***Primary caregiver***	
*Age of primary caregiver (e*.*g*. *father)*	40 (35–45)
*Education*	
Superior education, University	145 (14.2)
Superior education, not University	216 (21.1)
High School Certificate	603 (58.9)
Primary School or Literate	59 (5.8)
Illiterate	0 (0.0)
*Occupation*	
Retired, disability	10 (1.0)
Unemployed	16 (1.6)
Semi-skilled worker	565 (55.2)
Skilled worker	156 (15.2)
Technical career	137 (13.4)
Professional	139 (13.6)
***School***	
*Urban*	838 (81.9)
*Public*	440 (43.0)
***Distance to the nearest health care center***	
*<1 km*	493 (48.2)
*≥1 to <5 km*	435 (42.5)
*≥5 to <10 km*	80 (7.8)
*≥10 to <20 km*	13 (1.3)
*≥20 km*	2 (0.2)
***Most recent doctor visit***	
*In the last month*	236 (23.1)
*In the last 6 months*	366 (35.8)
*In the last year*	208 (20.3)
*In the last 2 years*	71 (6.9)
*More than 2 years ago*	142 (13.9)

**Table 2:** Physical examination.

Height for age z-score	-0.43±1.03
Weight for age z-score*	0.78±1.25
BMI (kg/m^2^) for age z-score	1.05±1.15
Body surface area (m^2^)	1.3 (1.0–1.5)
Waist circumference	71.0 (64.0–78.0)
Heart rate (bpm)	81 (74–85)
Oxygen saturation (%)	96 (95–97)
Cardiac murmur	44 (4.3)
*Systolic*	41 (4.0)
*Diastolic*	3 (0.3)
History of sore throat	394 (38.5)
Joint pain	286 (28.0)
Signs of ARF	
*Migrating arthritis*	2 (0.2)
*Erythema marginatum*	0 (0.0)
*Subcutaneous nodules*	0 (0.0)
*Chorea*	1 (0.1)

*z-scores for weight and age are only available up to the age of 120 months.

**Fig 2:**
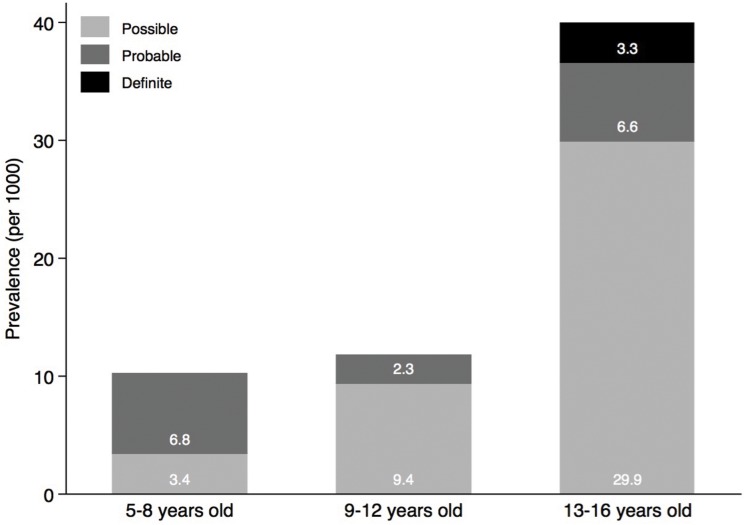
WHO stage of disease according to age categories from 5 to 8 years, 9 to 12 years and 13 to 16 years. Light grey indicated possible RHD, grey indicates probable RHD, and dark grey indicates definite RHD according to the modified WHO classification. The association between RHD according to the modified WHO classification and the age groups was assessed using the χ^2^ test (p = 0.010). The association between disease progression and age was evaluated using unweighted ordered logistic regression (p = 0.016) and weighted ordered logistic regression (p = 0.416).

After on-site screening, pathologic findings on echocardiography were reported in 59 children (5.8%), out of which 45 underwent a confirmatory echocardiogram (Fig 1). From the remaining, 2 refused further participation, 1 changed school with no contact details, and 11 missed at least three appointments. Echocardiographic findings are summarized in Table 3. 20 children had findings consistent with definite (n = 1), probable (n = 5) or possible (n = 14) RHD according to the modified WHO criteria, resulting in a prevalence of 19.7/1000 children. The sensitivity and specificity of a cardiac murmur to detect RHD were 25% and 96%, respectively. The prevalence of RHD as assessed by the WHO definition increased across age categories from 10.2/1000 among children 5 to 8 years of age to 39.8/1000 among children 13 to 16 years of age (p = 0.010), and stage of disease numerically increased with advancing age without reaching statistical significance (p = 0.704) (Fig 2). 4 Children had definite or borderline RHD according to the WHF criteria, consistent with a prevalence of 3.9/1000. Echocardiographic details of the WHF criteria are summarised in S3 Table. The prevalence adjusted and bias adjusted Kappa measuring the inter-rater reliability ranged between 0.82 and 0.95, whereas the observed agreement was between 91% and 98% (S4 Table). The burden of RHD was paralleled by the burden of structural congenital heart disease (Table 3). 15 children were found to have left-to-right shunts through atrial septal defects (ASD, n = 10), patent ductus arteriosus (PDA, n = 4) or partial anomalous pulmonary venous drainage (n = 1). Additional incidental findings included 4 bicuspid aortic valves, one double mitral valve orifice, and one left ventricular noncompaction. Four children had both RHD and congenital heart disease (two had bicuspid aortic valve, one partial anomalous pulmonary venous drainage, and one ASD). There was a significant association between congenital heart disease and RHD (p = 0.001). We performed both an unweighted and weighted logistic regression to assess predictors of RHD according to the modified WHO criteria (Table 4). The unweighted model revealed that increasing age is associated with disease (p = 0.016) (consistent with Fig 2). This finding was however not maintained in the weighted model.

**Table 3:** Echocardiographic findings.

**Rheumatic heart disease**	
***WHF criteria***	4 (0.4)
*Definite RHD*	1 (0.1)
Pathologic MR and at least two morphological criteria of RHD of the MV	0 (0.0)
MS mean gradient greater or equal 4 mmHg	0 (0.0)
Pathological AR and at least two morphological features of RHD of the AV	1 (100.0)
Borderline disease of both the AV and the MV	0 (0.0)
*Borderline RHD*	3 (0.3)
At least two morphological features of RHD of the MV without pathological MR or MS	1 (33.3)
Pathological MR	1 (33.3)
Pathological AR	1 (33.3)
***WHO criteria***	20 (2.0)
Definite RHD	1 (0.1)
Probable RHD	5 (0.5)
Possible RHD	14 (1.4)
**Congenital heart disease**	
Atrial septal defects	10 (1.0)
Partial anomalous pulmonary venous drainage	1 (0.1)
Bicuspid aortic valve	4 (0.4)
Double mitral valve orifice	1 (0.1)
Patent ductus arteriosus	4 (0.4)
Left ventricular noncompaction	1 (0.1)

MR = mitral regurgitation, MV = mitral valve, MS = mitral stenosis, AR = aortic regurgitation, AV = aortic valve.

**Table 4:** Predictors of rheumatic heart disease.

	Unweighted model		Weighted model	
	OR (95%CI)	P-value	OR (95%CI)	P-value
Age	1.24 (1.04–1.48)	0.0160	1.07 (0.87–1.32)	0.5326
Male gender	1.01 (0.42–2.44)	0.9894	1.63 (0.58–4.61)	0.3571
Number of household members	0.92 (0.74–1.14)	0.4628	0.85 (0.68–1.07)	0.1726
Overcrowding	1.20 (0.16–9.21)	0.8579	2.02 (0.26–15.95)	0.5058
Socio-economic score	1.03 (0.82–1.29)	0.7937	0.87 (0.66–1.15)	0.3194
History of sore throat	1.07 (0.43–2.63)	0.8903	1.60 (0.56–4.59)	0.3827

Management of patients with pathologic findings during screening echocardiography is summarized in S5 Table. Six children with WHO definite or probable RHD, and one with WHF borderline RHD were started on secondary antibiotic prevention with intramuscular benzathine penicillin. Three children with ASDs and right ventricular dilatation were referred for ASD closure; one additional ASD had already been treated by the time of confirmation. Six children with small ASDs were scheduled for regular clinical follow-up. Four children found to have PDAs were referred for percutaneous closure. All children with congenital heart defects were referred to specialized centres. Economic restrictions obliged the children to wait until the arrival of international missions who offer free interventions bringing their own materials, while using local infrastructure.[10]

## Discussion

The main findings of our study can be summarized as follows: (1) Prevalence of RHD varied according to definition applied and ranged from 3.9/1000 for WHF borderline/definite to 19.7/1000 for WHO possible/probable/definite. (2) Overall prevalence and stage of disease increased with advancing age. (3) The number of children diagnosed with congenital heart disease paralleled the number of children diagnosed with RHD. (4) There was a significant association between congenital heart disease and RHD. (5) At short-term, the major clinical consequences of echocardiographic screening were related to congenital heart disease rather than to RHD. The long-term effect of echocardiographic screening for RHD needs to be determined.

The observed prevalence of RHD differentiates Peru from truly endemic regions in sub-Saharan Africa, Southeast Asia, and Oceania where several-fold higher prevalence rates have been reported[3]. Data on prevalence of RHD in Latin America are scarce and range from 1.3/1000 (95% CI 0.3 to 5.9) as assessed by cardiac auscultation to 4.1/1000 (95% CI 2.4–7.1) as assessed by echocardiography^1^. In a predominantly urban setting in Peru, we documented a prevalence rate of 3.9 to 19.7/1000 depending on the definition applied. Prevalence according to the WHF criteria was hence comparable to the reported prevalence in low-risk non-Indigenous children in Australia[11]. Previous analyses suggested a higher prevalence of RHD in rural settings.[12] [13] Our findings of a higher prevalence of RHD according to the WHO compared to the WHF criteria are consistent with a previous report from Fiji.[14] While the modified WHO criteria are more sensitive, the echocardiographic criteria suggested by the WHF are more specific. Still, concerns have been raised that the WHF criteria may still potentially include some normal cases in the borderline group.[14] Follow-up studies of subclinical RHD reported stable findings in a majority of children and progression of disease in 5 to 15% only[15] [16] [17] [18]; however duration of follow-up was limited to less than 3 years and no long-term data is available to date. Another issue is related to the reliability of diagnosis of RHD during screening. The evaluation of the inter-rater agreement indicated differences in the detection of disease across the five off-site raters. Definition of RHD has important implications for the initiation of secondary prevention. Little is known about the natural course of early stages of RHD at this time. Regression has been observed in approximately one third of subclinical disease and was mainly attributable to regression of mitral regurgitation, whereas morphological changes tended to be more stable.[15] [16] [17] [19] Long-term studies will be needed to balance the benefit and risk of secondary antibiotic prevention for early stages of RHD. In view of the considerable inter-rater variability in echocardiographic detection of early lesions and documented regression of borderline disease, the burden of disease warranting population-based echocardiographic screening needs to be determined.

We observed an increasing prevalence of RHD as a function of age in an unweighted analysis. This observation is consistent with the findings of a meta-analysis on active surveillance of RHD and may underscore the effect of cumulative exposure to group A beta-hemolytic streptococcal infections.[3] None of the children in the youngest age category between 5 to 8 years was diagnosed with an advanced stage of disease. The number of children with definite or probable RHD numerically increased with advancing age without reaching statistical significance. Data from Senegal and Pakistan suggested an increasing prevalence of advanced stages of disease with increasing age.[20] [21]

Echocardiographic screening revealed congenital heart disease in 2.1% of children. Arequipa is situated at 2328 meters altitude above sea level. Previous studies have shown a higher prevalence of congenital heart disease in high altitude regions, particularly ASDs and PDAs.[22] [23] A higher prevalence of PDAs may result from the lower oxygen tension in higher altitudes, preventing the constriction and spontaneous closure of the ductus arteriosus after birth.[22] A higher prevalence of ASDs has been attributed to the higher pulmonary vascular resistance in altitude; the latter increases right-sided cardiac pressure inhibiting early closure of the foramen ovale. Prolonged stretching of the fossa ovalis during growth may in turn favour the formation of an ASD.[24] In addition to a higher prevalence of congenital heart disease at higher altitudes, the risk of congenital heart disease to be missed at higher altitudes also appears to be increased. Pulse oxymetry screening for cyanotic congenital heart disease among new-borns has shown a higher rate of failure at moderate altitude as compared to sea level due to lower mean oxygen saturations at higher altitude.[25] Moreover, economic restraints might prevent regular or detailed examinations of children. As a consequence, echocardiographic screening of school children may reveal congenital heart disease that could have been detected in postnatal exams or early childhood in wealthy countries.

The presence of congenital heart disease was associated with RHD in our study. This finding may be a chance finding; in view of the small numbers our analysis is not robust enough to postulate an association between RHD and congenital heart disease. Furthermore, a diagnosis of RHD has to be made with caution among subjects with congential heart disease involving the same structures that are also affected by RHD. Moreover, this observation may be biased by the fact, that children with suspicious findings for RHD may have undergone more detailed echocardiographic evaluation. This in turn may have increased the likelihood of being diagnosed with incidental congenital heart disease. However, the uniform application of a pre-specified comprehensive echocardiographic acquisition protocol including a rule-out list for congenital abnormalities reduces the chances of a selection bias. While the prevalence of congenital heart disease numerically paralleled the prevalence of RHD, the severity of disease was greater. Eight children required intervention for congenital heart disease whereas none of the children with RHD was found to be in a stage requiring intervention beyond secondary prevention. The short-term effect of screening was determined by collateral findings rather than RHD. As a consequence, strategies to address collateral findings from echocardiographic screening are necessary in the setup of active surveillance programs for RHD. The long-term effect of echocardiographic detection of clinically silent RHD needs to be determined in prospective studies.

The present analysis has several limitations. First, the sample size was limited, the number of children with RHD was small, and more than one quarter of children eligible for screening were not enrolled due to missing consent or repeated absence during screening visits. Moreover, screening was limited to school-going children. Since school attendance is associated with socio-economic status, sampling based on school lists may have further underestimated the burden of disease. Prevalence must therefore be interpreted with caution. However, schooling rates in Peru are well above 97%.[26] Second, the number of children with missing consent underlines the importance of adjustment to non-response. Adjustment to non-response might be responsible for the lack of statistical significance of the influence of age on RDH when sampling weights are considered because of unequal distribution of non-response across age categories and thus important differences in means of the sampling weights per age groups. This suggests that the withdrawal of the consent of participation is linked to age. Third, 14 out of 59 children (24%) with suspicious findings did not undergo confirmation. A similar drop-out rate has been observed in a previous study[18] and may questions the applicability of a two-step approach. Finally, estimated prevalence is bound to the setting under investigation and cannot be extrapolated to remote rural regions of Peru, or other regions in Latin America. And finally, the present findings on echocardiographic screening have to be interpreted in the context of a relatively low prevalence setting and cannot be generalized to endemic regions in sub-Saharan Africa, Southeast Asia, and Oceania.

## Supporting Information

S1 Fig Echocardiographic acquisition protocol checklist. (DOCX)

S1 Table Classifications of rheumatic heart disease. (DOCX)

S2 Table Number of students in sampling frame and study sample. (DOCX)

S3 Table Echocardiographic details according to WHF criteria. (DOCX)

S4 Table Interrater reliability. (DOCX)

S5 Table Diagnosis and management. (DOCX)
