# Supplementary material for: Screening for Rheumatic Heart Disease among Peruvian Children: A Two-Stage Sampling Observational Study
Source: PLoS One. 2015 Jul 24;10(7):e0133004. doi: 10.1371/journal.pone.0133004 (PMC4514892; doi:10.1371/journal.pone.0133004)
Supplement: S1 Fig — (DOCX) [file pone.0133004.s001.docx]

**S1 Fig. Echocardiographic acquisition protocol checklist**

Para-sternal long-axis view (PLAX)

Standard PLAX view (2D, loop)

Color Doppler (aortic valve [AV] and mitral valve [MV], loop)

Para-sternal short-axis view (PSAX)

Aortic valve, right ventricular outflow tract (RVOT) and pulmonary valve (PV) (2D, loop)

Pulmonary artery

Pulmonary regurgitation (PR) and rule-out of patent ductus arteriosus (color D., loop)

Trans-pulmonary continuous wave Doppler (CWD) (still image)

Pulsed-wave Doppler (PWD) in the pulmonary trunk (still image)

PWD in RVOT – just before PV leaflets (still image)

Short axis at mitral valve plane (2D, loop)

Mid-ventricular short axis (2D, loop)

Mid-ventricular short axis (M-mode, still image)

Apical 2 chamber view

Standard apical 2-CH view (loop)

Color Doppler (AR and MR, loop)

Apical 3 chamber view

Standard apical 3-CH view (loop)

Color Doppler (AR and MR, loop)

Apical 4 chamber view

Standard apical 4-CH view (2D, loop)

MAPSE (M-mode, still image)

Doppler color (AR and MR, loop) and rule-out of atrial and ventricular septal defects

CWD through MV (still image)

CWD through AV (still image)

PWD for LV filling (still image)

PWD at left ventricular outflow tract (still image)

DTI of the lateral annulus of the MV (loop)

DTI of the septal annulus of the MV (loop)

Apical 4 chamber view

Focus in the right ventricle (2D, loop)

TAPSE (M-mode, still image)

Color Doppler at the tricuspid valve (TV) (loop)

CWD through TV (still image)

PWD for RV filling (still image)

DTI of the RV free wall (loop)

Subcostal: Inferior cava vein (M-mode, loop) and rule-out of atrial septal defects

Suprasternal: Aortic arch (2D, loop)

CWD in descending aorta and rule-out of aortic coarctation
