# Supplementary material for: Screening for Rheumatic Heart Disease among Peruvian Children: A Two-Stage Sampling Observational Study
Source: PLoS One. 2015 Jul 24;10(7):e0133004. doi: 10.1371/journal.pone.0133004 (PMC4514892; doi:10.1371/journal.pone.0133004)
Supplement: S1 Table — (DOCX) [file pone.0133004.s002.docx]

| **S1 Table.** | **Classifications of rheumatic heart disease** |
| --- | --- |
| **Modified World Health Organization (WHO) criteria of rheumatic heart disease** | |
| - **Definite** | Cardiac murmur* AND significant mitral regurgitation and/or significant aortic regurgitation AND a thickened mitral valve and/or elbow deformity of the anterior mitral leaflet. |
| - **Probable** | Comes from a population in which RHD is endemic AND has a cardiac murmur* AND either significant mitral regurgitation and/or aortic regurgitation OR thickened mitral valve and/or elbow deformity of the anterior mitral leaflet. |
| - **Possible** | Comes from a population in which RHD is endemic, has no cardiac murmur, AND has thickened mitral valve and/or elbow deformity of mitral valve and/or significant mitral and/or aortic regurgitation. |
| **World Heart Federation (WHF) criteria for individuals aged ≤20 years** | |
| - **Definite** | 1. Pathological mitral regurgitation^†^ and at least two morphological features of RHD of the mitral valve^‡^ 2. Mitral stenosis mean gradient ≥4 mmHg 3. Pathological aortic regurgitation^#^ and at least two morphological features of RHD of the aortic valve^##^ 4. Borderline disease of both the aortic valve and mitral valve |
| - **Borderline** | 1. At least two morphological features of RHD of the mitral valve without pathological mitral regurgitation or mitral stenosis 2. Pathological mitral regurgitation 3. Pathological aortic regurgitation |
| - **Normal** | A) mitral regurgitation that does not meet all four Doppler echocardiographic criteria (physiological mitral regurgitation)  B) Aortic regurgitation that does not meet all four Doppler echocardiographic criteria (physiological aortic regurgitation)  C) An isolated morphological feature of RHD of the mitral valve (for example, valvular thickening) without any associated pathological stenosis or regurgitation  D) Morphological feature of RHD of the aortic valve (for example, valvular thickening) without any associated pathological stenosis or regurgitation |
| *Consistent with any combination of mitral regurgitation or aortic regurgitation  † Pathological mitral regurgitation: seen in two views AND jet length ≥2cm AND velocity ≥3 m/s for one complete envelope AND pan-systolic jet in at least one envelope  ‡ Morphological features of the mitral valve: Anterior mitral valve leaflet thickening ≥4mm; chordal thickening; restricted leaflet motion; excessive leaflet tip motion during systole  ^#^ Pathologic aortic regurgitation: seein in two views AND jet length ≥1cm AND velocity ≥3 m/s in early diastole AND pan-diastolic jet in at least one envelope  ^##^Morphological features of the aortic valve: irregular or focal thickening; coaptation defect; restricted leaflet motion; prolapse. | |
