# Supplementary material for: Screening for Rheumatic Heart Disease among Peruvian Children: A Two-Stage Sampling Observational Study
Source: PLoS One. 2015 Jul 24;10(7):e0133004. doi: 10.1371/journal.pone.0133004 (PMC4514892; doi:10.1371/journal.pone.0133004)
Supplement: S2 Table — (DOCX) [file pone.0133004.s003.docx]

| **S2 Table.** | Number of students in sampling frame and study sample | | | | |
| --- | --- | --- | --- | --- | --- |
|  |  | **Sampling frame** | | **Study sample** | |
|  |  | n (students) | Percentage | n (students) | Percentage |
| Urban | Public | 42973 | 28% | 429 | 31% |
|  | Private | 73589 | 48% | 669 | 48% |
| Rural | Public | 24666 | 16% | 173 | 12% |
|  | Private | 12235 | 8% | 124 | 9% |
