# Supplementary material for: Screening for Rheumatic Heart Disease among Peruvian Children: A Two-Stage Sampling Observational Study
Source: PLoS One. 2015 Jul 24;10(7):e0133004. doi: 10.1371/journal.pone.0133004 (PMC4514892; doi:10.1371/journal.pone.0133004)
Supplement: S3 Table — (DOCX) [file pone.0133004.s004.docx]

| **S3 Table.** | **Echocardiographic details according to WHF criteria** | | | |
| --- | --- | --- | --- | --- |
|  | | WHF Borderline/Definite RHD | No RHD |  |
| Number of children | | N=4 | N=999 |  |
| Mitral regurgitation (MR), n (%) | |  |  |  |
| Any MR | | 1 (25.0) | 83 (8.3) |  |
| MR seen in 1 view | | 0 (0.0) | 60 (6.0) |  |
| MR seen in ≥2 views | | 1 (25.0) | 22 (2.2) |  |
| MR jet length | |  |  |  |
| MR jet length of 1 cm to < 2 cm | | 1 (25.0) | 42 (4.2) |  |
| MR jet length ≥ 2 cm | | 1 (25.0) | 7 (0.7) |  |
| Presence of a pan-systolic jet of MR | | 1 (25.0) | 2 (0.2) |  |
| Aortic regurgitation (AR), n (%) | |  |  |  |
| Any AR | | 2 (50.0) | 7 (0.7) |  |
| AR seen in 1 view | | 0 (0.0) | 5 (0.5) |  |
| AR seen in ≥2 views | | 2 (50.0) | 3 (0.3) |  |
| AR jet length ≥ 1 cm | | 2 (50.0) | 4 (0.4) |  |
| AR jet velocity, m/s (IQR) | | 2 (50.0) | 1 (0.1) |  |
| Presence of a pan-diastolic jet of AR | | 2 (50.0) | 6 (0.6) |  |
| Mitral valve (MV) abnormality, n (%) | |  |  |  |
| AMVL thickness ≥4 | | 2 (50.0) | 16 (1.6) |  |
| Chordal thickening | | 2 (50.0) | 2 (0.2) |  |
| Restricted leaflet motion | | 0 (0.0) | 2 (0.2) |  |
| Aortic valve (AV) abnormality, n (%) | |  |  |  |
| Irregular or focal thickening | | 2 (50.0) | 1 (0.1) |  |
| Coaptation defect | | 3 (75.0) | 1 (0.1) |  |
