# Supplementary material for: Screening for Rheumatic Heart Disease among Peruvian Children: A Two-Stage Sampling Observational Study
Source: PLoS One. 2015 Jul 24;10(7):e0133004. doi: 10.1371/journal.pone.0133004 (PMC4514892; doi:10.1371/journal.pone.0133004)
Supplement: S4 Table — (DOCX) [file pone.0133004.s005.docx]

| **S4 Table. Interrater reliability** | | | | | |
| --- | --- | --- | --- | --- | --- |
| Rater | Kappa | Observed agreement | PABAK | Prevalence index | Bias index |
| Rater 1 | -0.020 | 0.958 | 0.917 | 0.958 | -0.008 |
| Rater 2 | -0.011 | 0.975 | 0.950 | 0.975 | 0.008 |
| Rater 3 | 0.129 | 0.908 | 0.817 | 0.892 | -0.075 |
| Rater 4 | 0.231 | 0.950 | 0.900 | 0.933 | -0.033 |
| Rater 5 | 0.159 | 0.925 | 0.850 | 0.908 | -0.058 |
