# Supplementary material for: Screening for Rheumatic Heart Disease among Peruvian Children: A Two-Stage Sampling Observational Study
Source: PLoS One. 2015 Jul 24;10(7):e0133004. doi: 10.1371/journal.pone.0133004 (PMC4514892; doi:10.1371/journal.pone.0133004)
Supplement: S5 Table — (DOCX) [file pone.0133004.s006.docx]

| **S5 Table.** | | | **Diagnosis and management** | | | |
| --- | --- | --- | --- | --- | --- | --- |
| Age (years) | Gender | Rheumatic heart disease | | | Congenital heart disease | Planned Management |
|  |  | WHO criteria | | WHF |  |  |
| 7 | Female | Probable | | Borderline |  | No treatment* |
| 11 | Female | Probable | | Borderline |  | Secondary prevention |
| 13 | Male | Probable | | Normal |  | Secondary prevention |
| 11 | Female | Possible | | Borderline |  | Secondary prevention |
| 11 | Male | Possible | | Normal |  | Follow-up |
| 14 | Female | Possible | | Normal |  | Follow-up |
| 9 | Male | Possible | | Normal |  | Follow-up |
| 13 | Female | Possible | | Normal |  | Follow-up |
| 13 | Male | Possible | | Normal |  | Follow-up |
| 7 | Female | Possible | | Normal |  | Follow-up |
| 11 | Female | Possible | | Normal |  | Follow-up |
| 14 | Male | Possible | | Normal |  | Follow-up |
| 14 | Female | Possible | | Normal |  | Follow-up |
| 14 | Female | Possible | | Normal |  | Follow-up |
| 15 | Male | Possible | | Normal |  | Follow-up |
| 15 | Female | Possible | | Normal |  | Follow-up |
| 13 | Male | Definite | | Definite | Bicuspid aortic valve | Secondary prevention |
| 7 | Male | Probable | | Normal | Partial anomalous pulmonary venous drainage | Secondary prevention, surgical correction |
| 15 | Male | Probable | | Normal | ASD with dilated RV | Secondary prevention, s/p ASD closure** |
| 15 | Male | Possible | | Normal | Bicuspid aortic valve | Follow-up |
| 10 | Male |  | |  | ASD with dilated RV | s/p ASD-closure*** |
| 12 | Male |  | |  | ASD with dilated RV | ASD-closure |
| 13 | Male |  | |  | ASD with dilated RV | ASD-closure |
| 8 | Female |  | |  | ASD with normal RV | Follow-up |
| 10 | Male |  | |  | ASD with normal RV | Follow-up |
| 10 | Female |  | |  | ASD with normal RV | Follow-up |
| 11 | Female |  | |  | ASD with normal RV | Follow-up |
| 10 | Female |  | |  | ASD with normal RV | Follow-up |
| 11 | Female |  | |  | ASD with normal RV | Follow-up |
| 13 | Male |  | |  | Bicuspid aorta | Follow-up |
| 15 | Male |  | |  | Bicuspid aorta | Follow-up |
| 11 | Female |  | |  | Double mitral valve orifice | Follow-up |
| 14 | Female |  | |  | Left ventricular noncompaction cardiomyopathy | Follow-up |
| 9 | Female |  | |  | Patent ductus arteriosus | PDA closure |
| 7 | Female |  | |  | Patent ductus arteriosus | PDA closure |
| 10 | Female |  | |  | Patent ductus arteriosus | PDA closure |
| 14 | Female |  | |  | Patent ductus arteriosus | PDA closure |
|  |  |  | |  |  |  |
| * Patient treated previously with radiotherapy due to a hematologic disorder, which was considered a reasonable cause for valvular disease with no antibiotics prescribed. | | | | | | |
| ** Patient treated percutaneously before screening. | | | | | | |
| *** Patient treated surgically as a consequence of screening, one week after confirmation. | | | | | | |
